# Effects of a Virtual Reality Cycling Platform on Lower Limb Rehabilitation in Patients With Ataxia and Hemiparesis: Pilot Randomized Controlled Trial

**DOI:** 10.2196/39286

**Published:** 2024-01-04

**Authors:** Ana Rojo, Arantxa Castrillo Calvillo, Cristina López, Rafael Raya, Juan C Moreno

**Affiliations:** 1 Departamento de Tecnologías de la Información, Escuela Politécnica Superior, Universidad San Pablo-CEU, CEU Universities, Madrid, Spain; 2 Neural Rehabilitation Group, Spanish National Research Council, Cajal Institute, Madrid, Spain; 3 Centro Lescer, Madrid, Spain

**Keywords:** ataxia, cycling, hemiparesis, lower limb, neuropathology, rehabilitation, virtual reality, limb, intervention, neural, neural plasticity, therapy, muscle, strength, balance, tool, exercise, physical activity, neuroplasticity

## Abstract

**Background:**

New interventions based on motor learning principles and neural plasticity have been tested among patients with ataxia and hemiparesis. Therapies of pedaling exercises have also shown their potential to induce improvements in muscle activity, strength, and balance. Virtual reality (VR) has been demonstrated as an effective tool for improving the adherence to physical therapy, but it is still undetermined if it promotes greater improvements than conventional therapy.

**Objective:**

Our objective was to compare the effect on lower limb range of motion (ROM) when using VR technology for cycling exercise versus not using VR technology.

**Methods:**

A randomized controlled trial with 20 patients with ataxia and hemiparesis was carried out. The participants were divided into 2 groups: the experimental group (n=10, 50%) performed pedaling exercises using the VR system and the control group (n=10, 50%) performed pedaling exercises without using VR. Measurements of the active and passive ROM of the hip and knee joint were taken before and after a cycling intervention, which consisted of 3 sessions of the same duration but with progressively increasing speeds (4, 5, and 6 km/h). Repeated measures ANOVAs were conducted to compare the preintervention (T_i_) and postintervention (T_e_) assessments within each group. Additionally, the improvement effect of using the VR system was analyzed by comparing the variation coefficient (*Δ* = 1 – [T_e_ / T_i_]) between the preintervention and postintervention assessments for each group. Group comparisons were made using independent 1-tailed *t* tests.

**Results:**

Significant improvements were shown in active left hip flexion (*P*=.03) over time, but there was no group-time interaction effect (*P*=.67). Passive left hip flexion (*P*=.93) did not show significant improvements, and similar results were observed for active and passive right hip flexion (*P*=.39 and *P*=.83, respectively). Neither assessments of knee flexion (active left: *P*=.06; passive left: *P*=.76; active right: *P*=.34; passive right: *P*=.06) nor knee extension showed significant changes (active left: *P*=.66; passive left: *P*=.92; active right: *P*=.12; passive right: *P*=.38). However, passive right knee extension (*P*=.04) showed a significant improvement over time. Overall, although active and passive ROM of the knee and hip joints showed a general improvement, no statistically significant differences were found between the groups.

**Conclusions:**

In this study, participants who underwent the cycling intervention using the VR system showed similar improvement in lower limb ROM to the participants who underwent conventional training. Ultimately, the VR system can be used to engage participants in physical activity.

## Introduction

### Background

Ataxia is an umbrella term for describing deficits in limb movement coordination such as dysmetria, dyspraxia, and dyssynergia [1]. The persistence of these deficits affects an individual’s functional ability and poses a health challenge for both patients and clinicians.

Current scientific evidence indicates that the most effective treatment for ataxia should combine balance and coordination retraining and constraint-induced functional movement therapy [2]. However, the scientific literature still lacks a consensus on the details of these interventions and the timing of their implementation to enhance the recovery of the functionality of motor deficits in an individual [3].

On the other hand, in the field of neurophysiology, it is well known that to induce changes in neuroplasticity to achieve the functional recovery of motor deficits, the application of therapies based on the repetition of movements is required [4]. Some studies point out that the principles of motor learning are directly related to the regeneration of structures and the reorganization of neuronal function [5,6]. Moreover, the amount of practice is a key factor in motor learning, as well as the feedback provided during practice [7]. In fact, physical therapists must consider both the error feedback and activity guidance as 2 fundamental components of patient interaction during therapy to promote neuromotor learning [8]. Thus, interventions that promote normal function rather than the compensation of deficits are more recommended and should be applied to generate a physical activity plan based on the principles of motor learning and neural plasticity for patients with ataxic hemiparesis.

### Prior Work

The scientific literature in the field of neurorehabilitation shows that pedaling exercises have the potential to induce improvements in muscle activity, strength, and balance [9]. This is mainly due to the fact that pedaling exercises based on the use of a cycloergometer provide a high number of flexion and extension repetitions [10] in the lower extremities for considerable periods of time. Because pedaling and walking are cyclical locomotor tasks that require the lower limb to alternate between flexion and extension [11,12], both share similar locomotor patterns of alternating muscle activation of antagonists [10,13]. Thus, cycling exercises are found to be useful for strengthening the lower limb muscles while acting as a pseudowalking task-oriented exercise. Some studies eluded that those biomechanical functions may be altered by the muscle groups involved in the pedaling tasks [14-16]. In fact, it was found that the degradation of pedaling performance in adults with hemiparesis was related to abnormalities in the execution of specific biomechanical functions [15]. Subsequently, it has been proven that human walking and cycling shared similar muscle synergies [16]. This evidence is the basis for rehabilitation treatments based on pedaling movements with potential positive outcomes for walking [16].

The ergometer is an equipment designed to perform cardiovascular work based on the alternative circular movement of the lower limb. Its use is advantageous for a muscle coordination study because balance is not an applicable factor in this kinematically constrained task [13]. In fact, applying an ergometer-based cycling routine could be useful because it requires no balance. Moreover, the exercise intensity of the ergometer-based cycling can be adapted to the user by adjusting the resistance of the pedal or the target speed. The ability to personalize the intensity of the exercise is a relevant factor for the patient’s rehabilitation process. For these reasons, regular ergometer-based cycling is found to be a safer unsupervised exercise that is recommended for lower limb rehabilitation. Nevertheless, cycling exercise is also a static and repetitive form of exercise that leads to boredom and listlessness in patients. To deal with this discouragement factor, emerging technologies have been applied to elicit intrinsic motivation for rehabilitation patients [17]. Several studies pointed out the usefulness of gaming elements and virtual environments as assistive technology [18,19] and their potential effectiveness in physical therapies as opposed to conventional therapies [20].

Quite a few studies have focused on the analysis of functional metrics in virtual pedaling. A recent study evaluated the functionality of a virtual reality (VR) cycling training program that was applied to 10 patients with stroke [21]. It assessed the improvement of the bilateral asymmetry between the experimental group and the control group after the VR cycling intervention program. To evaluate this index, they equipped the ergometer pedals with force plates to determine the effect of the VR cycling training on each limb. The improvement of bilateral strength and standing balance was significantly different between VR cycling training and traditional physical training. Similarly, a previous study compared the effects of a cycling training program with extrinsic biofeedback and a nonimmersive interface versus traditional physical training on lower limb functional recovery in patients with stroke [22]. The results showed that improvements in walking endurance, walking speed, and muscle spasticity of the group using VR were significantly better than the group who underwent traditional physical training.

### Objectives

The main objective of this study was to evaluate 2 different interventions: pedaling with VR and pedaling without VR. This study focused on comparing the improvements in lower limb range of motion (ROM) in pedaling activity between the group using VR and the group not using VR. To this end, a randomized controlled trial was carried out with patients with ataxia and hemiparesis. Hip and knee ROMs were measured before and after the cycling intervention. The overall aim of these analyses was to determine the effects of the 2 different interventions on short-term improvement of lower limb function and ROM.

## Methods

### VR System

The VR system implements extrinsic feedback strategies, gamification by levels, and personalization of the sessions with the aim of achieving greater adherence to pedaling exercise sessions. Its immersive nature means an increase in the sense of “presence,” promoting the active involvement of the user. The VR system is based on the transmission of the cycling kinematic data captured by the inertial sensors to the Oculus Quest 2 (Meta) head-mounted display (HMD) via Bluetooth. Therefore, the virtual application estimates the pedaling cycles, cadence, and distance during the exercise activity. The VR scenarios generated for this therapy consist of mapping the cycling cadence to the vehicle speed. Thus, the patient is placed inside a vehicle and visualizes the session data on the control panel while moving at the speed of the pedaling motion.

The design of the VR experience has been technically validated computationally to ensure low latency in motion analysis and visual representation of motion [23], thus preserving the embodiment effect and the sense of presence. Subsequently, the platform has also been validated from the point of view of satisfaction and ease of use of the system [24]. Additionally, considering that it is a stationary experience with an HMD that simulates a displacement, we evaluated to which extent the VR experience generates the type of motion sickness that causes fatigue, nausea, disorientation, postural instability, or visual fatigue [25]. Indeed, we verified that the platform does not generate adverse effects due to cybersickness [24].

### Recruitment

**Figure 1.**
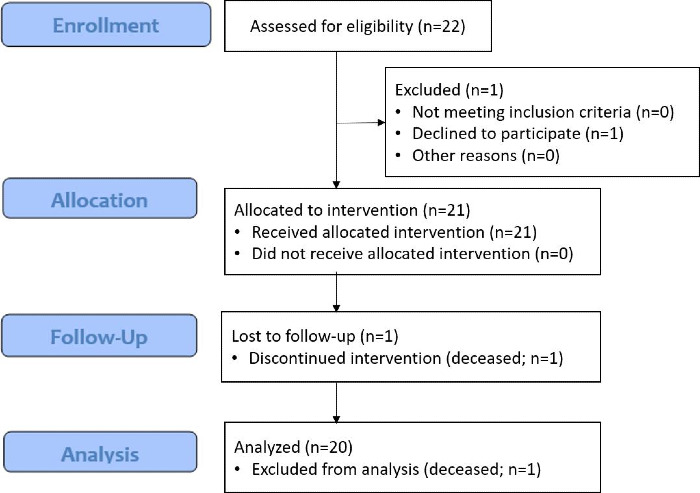
CONSORT (Consolidated Standards of Reporting Trials) diagram.

The participants were patients of both sexes between 18 and 90 years of age, recruited at the Lescer Clinic applying the inclusion and exclusion criteria. Inclusion criteria were as follows: individuals were eligible if they (1) had been prescribed pedaling exercise as treatment for lower limb rehabilitation and (2) were able to perform a pedaling session with VR technology. Exclusion criteria were as follows: (1) an insufficient cognitive state, (2) an unbound bone fracture, (3) severe disorders of vision or audition (inability to perceive visual or auditory information coming from VR), and (4) any incompatibility with the use of a VR system according to the clinical record. A sample of 22 participants (n=13, 59% male and n=7, 32% female; mean age 59.90, SD 13.56 y) volunteered to participate in this pilot randomized controlled trial (Table 1). Of this 22-person cohort, 1 participant dropped out of the study and 1 participant did not complete the study (Figure 1). The cohort was randomly divided into the experimental group (EG; 9/10, 90% male and 1/10, 10% female; mean age 60.80, SD 12.26 y) with VR cycling exercises or the control group (CG; 4/10, 40% male and 6/10, 60% female; mean age 59.00, SD 14.69 y) with traditional cycling exercises.

**Table 1.** Clinical and epidemiological features of the experimental group (EG) and control group (CG) participants.

Group and participant number		Sex	Age (y)	Etiology	Condition
**EG**					
	1	Male	57	Ischemic stroke	Hemiparesis
	2	Male	71	Hemorrhagic stroke	Ataxia
	3	Male	53	Hemorrhagic stroke	Ataxia
	4	Male	72	MCA^a^ stroke	Hemiparesis
	5	Male	53	MCA stroke	Hemiparesis
	6	Male	62	Ischemic stroke	Hemiparesis
	7	Male	59	Hemorrhagic stroke	Ataxia
	8	Male	56	Progressive multifocal leukoencephalopathy	Ataxia
	9	Female	86	Hemorrhagic stroke	Hemiparesis
	10	Male	39	Ischemic stroke	Hemiparesis
**CG**					
	1	Male	45	MCA stroke	Hemiparesis
	2	Female	64	Hemorrhagic stroke	Ataxia
	3	Male	58	Guillain-Barré syndrome	Hemiparesis
	4	Female	41	Hemorrhagic stroke	Ataxia
	5	Female	49	Ischemic stroke	Ataxia
	6	Male	83	Ischemic stroke	Ataxia
	7	Female	80	Hemorrhagic stroke	Hemiparesis
	8	Female	72	Traumatic brain injury	Hemiparesis
	9	Male	57	Ischemic stroke	Hemiparesis
	10	Female	41	Guillain-Barré syndrome	Ataxia

a MCA: middle cerebral artery.

### Ethical Considerations

Ethical approval was obtained from the Research Ethics Committee of the San Pablo CEU University (550/21/51). This study has been registered at ClinicalTrials.gov (NCT05162040). All the participants were given written information in accordance with the Research Ethics Committee. The informed consent and the ability for participants to opt out was provided. Additionally, participants were informed that the data collected in this study can only be used for this study, not for secondary studies. The approval of the Research Ethics Committee of San Pablo CEU University only covers this study and does not cover a secondary analysis without additional consent. However, no additional analysis had been carried out.

To ensure privacy and confidentiality, data are collected by employees of the agencies participating in the study. Each participant is assigned a unique code along with personal sociodemographic data and informed consent. These files remain in the custody of the principal investigator in charge of the project, while the assigned number is the one that identifies the anonymized data that was later analyzed. Finally, the participation in this study is completely voluntary; no compensation of any nature is offered to the human participants.

### Intervention

This study was designed as a randomized controlled trial with 20 participants divided into 2 groups, following a block randomization method. The participants of the EG (n=10) performed pedaling exercises while using the VR system, whereas the participants of the CG (n=10) performed pedaling exercises without using the VR system. Before and after completing the exercise program, measurements of gait function metrics and joint ranges were performed to assess the effect of using VR stimulus during the cycling exercises.

**Figure 2.**
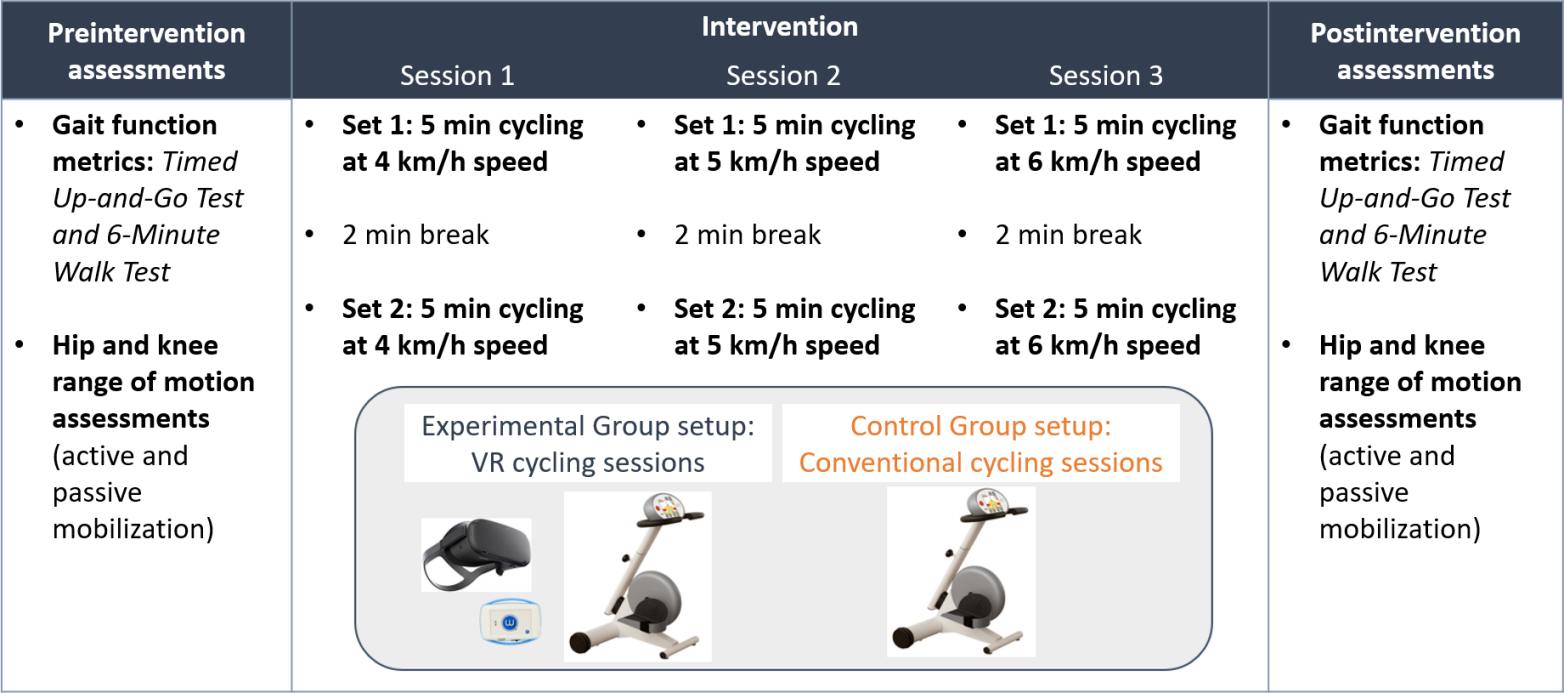
Summary of the intervention program for experimental and control group participants. VR: virtual reality.

The participants completed the cycling intervention simultaneously with their rehabilitation sessions. Afterward, for each participant, 3 cycling sessions were scheduled over 1 week with a maximum of 48 hours between sessions. Each session consisted of 2 sets of a 5-minute pedaling exercise spaced with a 2-minute break (to rest). Similar studies [19,26] have tested robotic unicycles in pedaling sessions at a cadence of 60 revolutions per minute. In our case, the pedaling speed of 1 cycle per second is equivalent to a target speed of 6 km/h. For this reason, it was decided to set this speed as the maximum speed and to start the first session with a slightly more comfortable speed (4 km/h) and increase it progressively (Figure 2). The participants of both groups performed the exercise following a set pedaling speed so that they received visual feedback according to the set target speed of 4-6 km/h for each session. The EG participants received visual feedback through the immersive VR application, whereas the CG participants received visual feedback on the ergometer display. All participants were instructed to maintain a constant pedaling speed throughout the session at the target cadence.

### Physical Assessment

For the assessment of active and passive ROM of the hip and knee joint, a specifical ROM assessment tool was used. Measurements were extracted from biomechanical analysis using an inertial motion capture system (Werium; Werium Solutions) consisting of 2 inertial sensors: 1 placed in the distal part of the extremity (moving sensor) and the other in the proximal part (fixed sensor). Both sensors send their measurements via Bluetooth to a PC that runs the data acquisition software, Pro Motion Capture (Werium Solutions). This software computes the relative angle from both angle measurements (avoiding compensations) with an accuracy of 1 degree.

### Protocol

**Figure 3.**
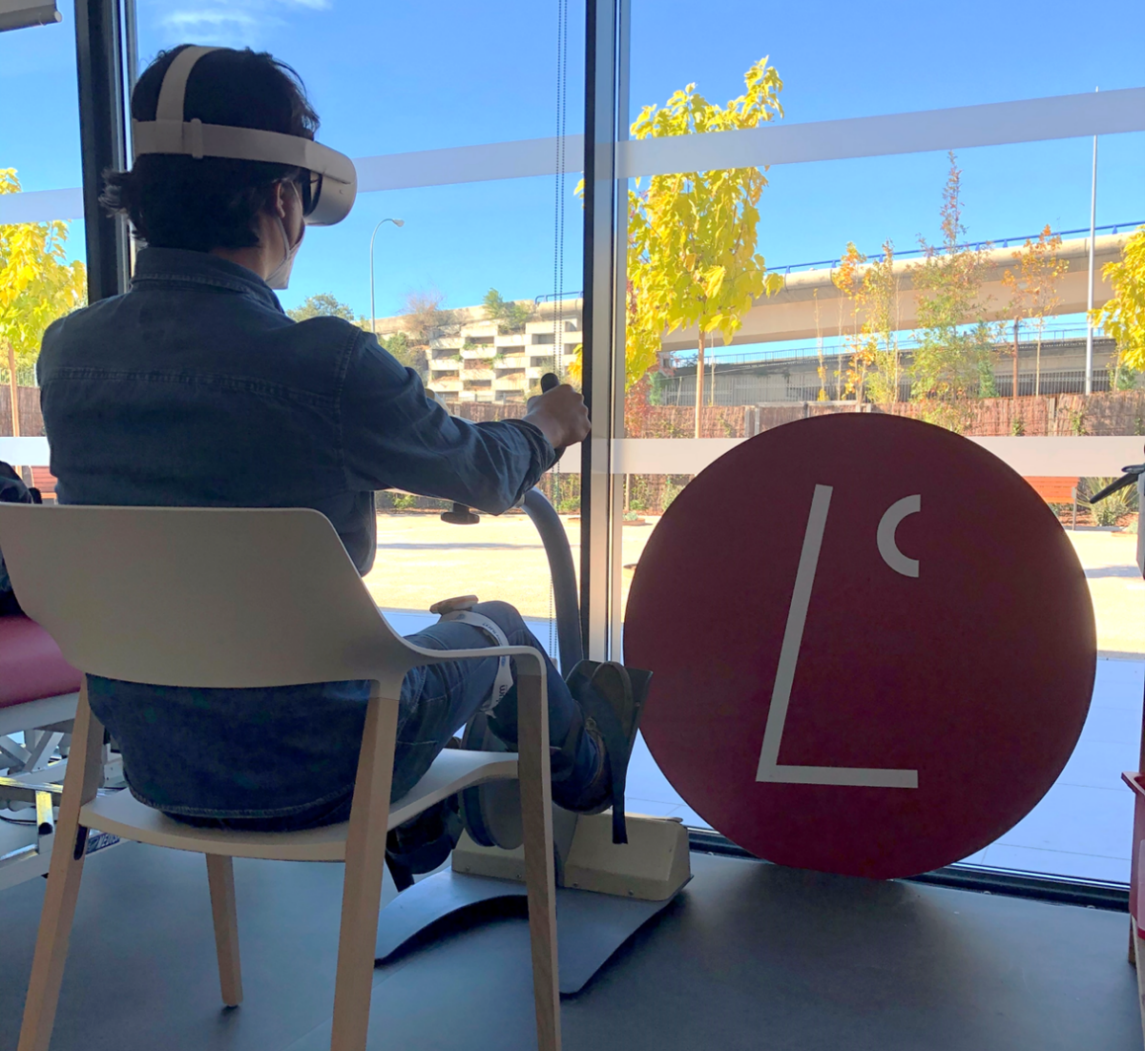
Cycling session of a participant in the experimental group using the virtual cycling platform.

The cycling sessions for both groups consisted of the use of a leg ergometer that allows training of the lower limb. Additionally, the EG used an inertial sensor placed on the right thigh and the Oculus Quest 2 HMD (Figure 3).

The EG underwent the following procedure each session:

- The clinician connected the inertial sensor to the Oculus Quest 2 HMD.
- The patient was seated in a nonmovable chair (with no armrests) in front of the pedaling station during the entire session. The inertial sensor was placed on the right thigh of the patient by adjusting an elastic band, and the sensor was turned on.
- The clinician fitted the Oculus Quest 2 HMD comfortably on the patient and guided him or her through the selection of the game scene. Once the game environment was entered, the clinician indicated the number of minutes of exercise and the target speed of the session so that the patient could configure these parameters on the interactive settings panel.
- Finally, the user performed 2 sets of a 5-minute cycling exercise with a 2-minute break between the sets.

Similarly, the CG underwent the following procedure each session:

- The patient was seated in a nonmovable chair (with no armrests) in front of the pedaling station during the entire session.
- The clinician turned on the ergometer’s display and entered the number of minutes of exercise and the target speed of the session.
- Finally, the user performed 2 sets of a 5-minute cycling exercise with a 2-minute break between the sets.

### Statistical Analysis

The data analysis model is the repeated measures model between 2 groups and the analysis of the longitudinal effect in increments of the measurements. Multifactor ANOVA analysis (with *P*<.05) were computed with SPSS Statistics (version 27.0; IBM Corp). The sample size was calculated using the software tool G*Power (version 3.1.9.7; Heinrich Heine Universität Düsseldorf). Ideally, assuming an effect size of 0.7, a minimum sample of 20 participants was required for the study to provide consistent statistical results. Since the effect size shows the strength of the relationships, it represents a minimum clinically meaningful difference. Of the many different types of effect sizes, the G*Power software uses Cohen *d* to characterize effect size by relating the mean difference to variability. Therefore, his study standardized the effect size to 0.7 for sample size calculation and power analysis.

## Results

To identify the underlying differences between the preintervention (T_i_) and postintervention (T_e_) assessments in each group, repeated measures ANOVAs were conducted with time (T_i_ – T_e_) as the dependent variable and group as the main within-subjects factor. When the ANOVA was significant, the Bonferroni post hoc test was used. To ensure that the error variance of the dependent variables is equal across groups, the Levene test was applied beforehand for all the metrics.

**Figure 4.**
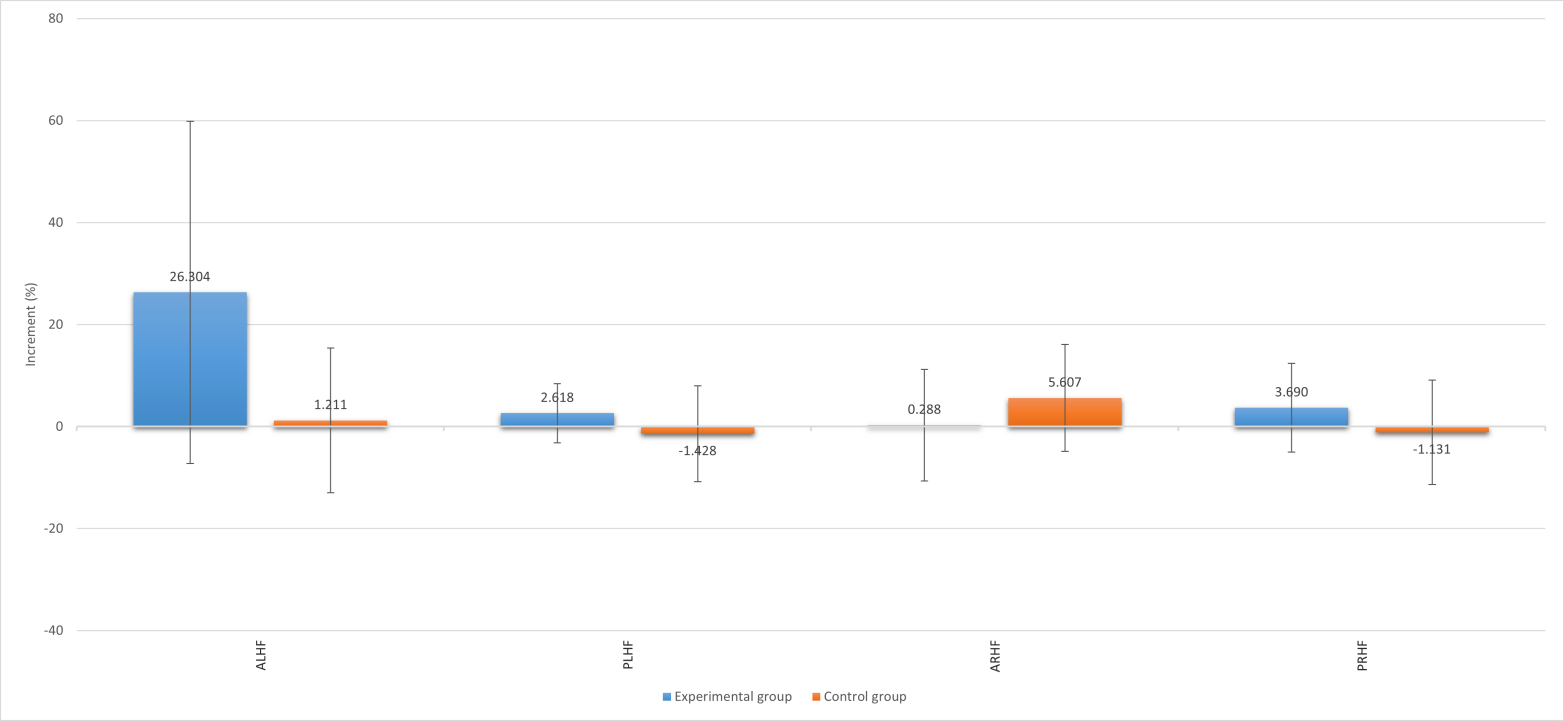
Summary of increments in active and passive hip ROM parameters with SD bars. The vertical axis represents the percentage of postintervention increase or decrease of each hip ROM parameter. ALHF: active left hip flexion; ARHF: active right hip flexion; PLHF: passive left hip flexion; PRHF: passive right hip flexion; ROM: range of motion.

In addition, to identify the improvement effect due to the use or nonuse of the VR system, the variation coefficient between the preintervention and postintervention assessments was analyzed for each group as follows: *Δ* = 1 – (T_e_ / T_i_). The variation coefficient outcomes were compared between groups by the independent 1-tailed *t* test. The mean and SD of the ROM outcomes for the hip and knee of each group are shown in Table 2. The mean increase *Δ* for each measurement is shown in Figures4  5.

**Table 2.** Hip and knee range-of-motion outcomes.

Outcome	Experimental group, mean (SD)			Control group, mean (SD)		
	Preintervention (°)	Postintervention (°)	Variation coefficient (%)	Preintervention (°)	Postintervention (°)	Variation coefficient (%)
ALHF^a^	81.25 (36.09)	94.23 (32.26)	26.30 (33.52)	92.84 (21.40)	94.37 (25.83)	1.21 (14.20)
PLHF^b^	106.07 (21.16)	107.94 (17.63)	2.61 (5.81)	112.92 (17.76)	110.70 (16.83)	–1.43 (9.40)
ARHF^c^	97.55 (20.94)	97.13 (21.26)	0.28 (10.94)	97.11 (28.05)	101.79 (27.35)	5.60 (10.50)
PRHF^d^	106.63 (17.06)	109.82 (14.99)	3.69 (8.72)	119.74 (14.73)	117.71 (13.42)	–1.13 (8.72)
ALKF^e^	46.07 (14.62)	45.97 (11.47)	4.27 (26.31)	37.47 (12.03)	35.65 (8.47)	1.63 (30.86)
PLKF^f^	58.82 (9.84)	55.96 (9.79)	–3.48 (17.40)	57.14 (13.92)	54.58 (12.15)	–1.66 (19.96)
ARKF^g^	39.13 (16.54)	37.81 (10.68)	8.98 (35.88)	43.03 (10.00)	44.58 (13.32)	5.36 (30.29)
PRKF^h^	50.57 (10.02)	49.81 (10.31)	–0.65 (15.15)	63.35 (12.28)	57.28 (13.95)	–9.35 (15.17)
ALKE^i^	61.72 (14.86)	62.92 (13.11)	3.28 (9.74)	55.57 (17.13)	63.41 (11.77)	26.70 (51.68)
PLKE^j^	66.46 (11.74)	69.95 (15.09)	4.94 (15.92)	64.75 (11.94)	72.30 (12.46)	14.91 (24.40)
ARKE^k^	64.00 (10.11)	68.02 (10.14)	8.33 (20.67)	57.49 (14.91)	57.19 (14.76)	2.22 (23.52)
PRKE^l^	66.67 (11.53)	67.18 (10.93)	1.58 (14.10)	57.65 (11.21)	68.78 (6.67)	25.29 (34.70)

a ALHF: active left hip flexion.

b PLHF: passive left hip flexion.

c ARHF: active right hip flexion.

d PRHF: passive right hip flexion.

e ALKF: active left knee flexion.

f PLKF: passive left knee flexion.

g ARKF: active right knee flexion.

h PRKF: passive right knee flexion.

i ALKE: active left knee extension.

j PLKE: passive left knee extension.

k ARKE: active right knee extension.

l PRKE: passive right knee extension.

**Figure 5.**
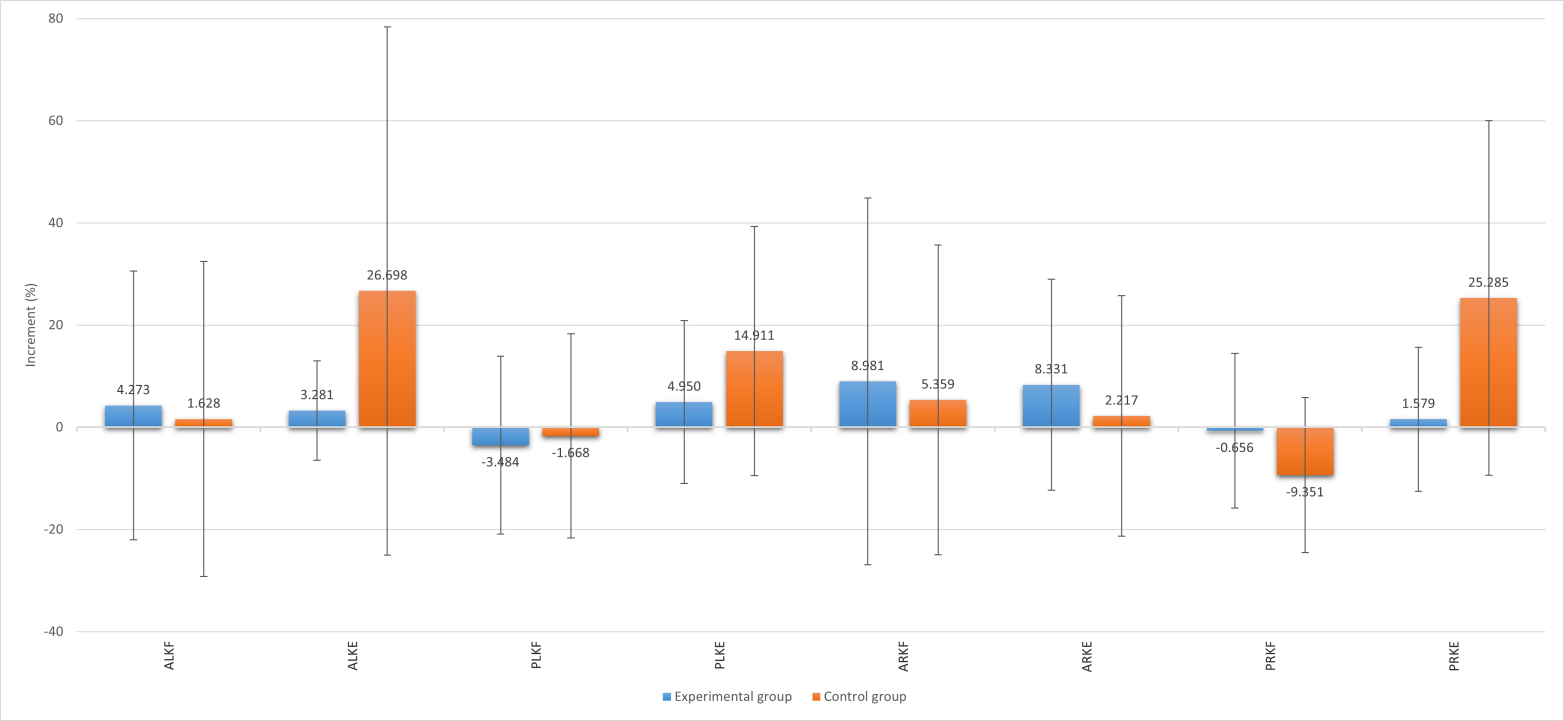
Summary of increments in active and passive knee ROM parameters with SD bars. The vertical axis represents the percentage of postintervention increase or decrease of each knee ROM parameter. ALKE: active left knee extension; ALKF: active left knee flexion; ARKE: active right knee extension; ARKF: active right knee flexion; PLKE: passive left knee extension; PLKF: passive left knee flexion; PRKE: passive right knee extension; PRKF: passive right knee flexion; ROM: range of motion.

With regard to the hip flexion outcomes, the active left hip flexion results were significant by ANOVA (*P*=.03), with no significance observed for the between-subjects effects test (*P*=.67). However, the within-subjects effects test was significant for the time factor (*P*=.03), but no significant group-time interaction effect was found (*P*=.08). Despite the opposing results showing passive left hip flexion improvements for each group, there was no significance difference by ANOVA (*P*=.93) and no statistically significant result was obtained by the between-subjects effects test. Passive left hip flexion was statistically significant in the within-subjects effects test for the time factor (*P*=.008). The active and passive right hip flexion results were not significant by ANOVA (*P*=.39 and *P*=.83, respectively). In both cases, no significant results were obtained for the between- and within-subjects effects tests.

For the knee ROM measurements, when analyzing the left knee assessments, the active and passive left knee flexion outcomes were not significant by ANOVA (*P*=.06 and *P*=.76, respectively). No statistically significant results were obtained by the between- and within-subjects effects tests in both cases. Similar results were obtained for the active left knee extension outcomes. Although reasonable differences in the active and passive left knee extension increases between groups can be observed in Figure 5, neither active nor passive left knee extension were significant by ANOVA (*P*=.66 and *P*=.92, respectively). No statistically significant results were obtained by the between- and within-subjects effects tests in both cases.

Regarding the right knee assessments outcomes, all outcomes were not significant by ANOVA (active flexion: *P*=.34; passive flexion: *P*=.06; active extension: *P*=.12; passive extension: *P*=.38). No statistically significant results were obtained by the between- and within-subjects effects tests for all cases, except for passive right knee extension, which was statistically significant for the time factor (*P*=.04) by the within-subjects effects test.

## Discussion

### Principal Findings

The aim of this study was to test the short-term effects of 2 different interventions on short-term improvement of lower limb function and ROM. For this purpose, a randomized controlled trial was carried out with participants with ataxia and hemiparesis.

In this study, the improvement outcomes of active and passive knee and hip joint ROMs due to the use of VR technology were inconclusive. Likewise, no statistically significant differences in the results between groups can be indicated. Even so, all the active ROMs measured—that is, performed by the patients—showed an increase with respect to the initial values. A greater disparity was observed in the passive measurements, although this may be attributed to the different passive mobilizations performed at each time by different physiotherapists. In this case, the active measurement is of special relevance in clinical terms because it indicates a ROM that the patient is able to achieve autonomously. On the other hand, large SDs in outcome variables clearly indicate that the improvements in the functional gait outcomes are not entirely consistent or represent a group effect. We observe that no significant effect can be attributed to VR intervention based on the statistical analysis of the immediate effects on gait function and joint ROM.

However, considering this similarity between groups, it can be pointed out that the use of VR has similar positive effects as the use of the conventional pedaling treatment. Thus, this immediate observation of effects leads us to conclude that the use of VR during pedaling exercise has similar effects to non-VR exercise training. Therefore, given that the use of VR technology does not worsen the improvement of lower limb ROM, and in line with the scientific literature [17-20], it may be advantageous to use it to maintain the patient’s motivation.

### Strengths and Limitations

A limitation of this study is the short-term nature of the intervention program. It is arguable that a longer intervention program would have shown more notable effects on functional improvement. However, assuming that it is precisely the treatment time that is one of the main causes of progress in physical improvement, the motivational impact of VR technology over time would need to be assessed. Therefore, further studies on the motivational impact of VR cycling versus conventional cycling on long-term physical activity remain to be addressed. Regarding these future studies, we suggest that cohort studies should be conducted among a population with more homogeneous neurological conditions. This recommendation is based on the limitations encountered in this study, where the difficulty of drawing conclusions about group changes or improvements with such wide SDs is presumably a reflection of the heterogeneity of the group.

Another factor to consider is that different physiotherapists were involved in taking the ROM measurements of the participants, although the measurement system was the same. This fact could be considered in future studies to evaluate interrater effects.

### Future Directions

We consider it relevant to analyze, in future studies, whether these improvements in active and passive ROM are accompanied by greater muscle activation, in particular, the hamstrings, rectus femoris, gastrocnemius, and tibialis anterior muscles, as suggested by scientific literature [27].

### Conclusions

The results of this trial demonstrate that pedaling exercises coordinated with VR technology works as successfully as conventional training for patients with lower limb disorders such as ataxia and hemiparesis. In this study, it was found that participants who performed the pedaling exercise program using the VR system showed similar results to the participants who performed the exercise activity without using VR technology. Overall, VR technologies can be a useful tool to help patients with ataxia and hemiparesis engage in lower limb exercise therapies.

## Supplementary material

10.2196/39286 Checklist 1 CONSORT-EHEALTH (Consolidated Standards of Reporting Trials of Electronic and Mobile Health Applications and Online Telehealth) checklist (V 1.6.1).

## Abbreviations

CG: control group
EG: experimental group
HMD: head-mounted display
ROM: range of motion
VR: virtual reality
